# Electronic Couplings and Conversion Dynamics between Localized and Charge Transfer Excitations from Many-Body Green’s Functions Theory

**DOI:** 10.1021/acs.jctc.4c00142

**Published:** 2024-05-21

**Authors:** Gianluca Tirimbò, Björn Baumeier

**Affiliations:** † Department of Mathematics and Computer Science, Eindhoven University of Technology, P.O. Box 513, 5600 MB Eindhoven, The Netherlands; ‡ Institute for Complex Molecular Systems, Eindhoven University of Technology, P.O. Box 513, 5600 MB Eindhoven, The Netherlands

## Abstract

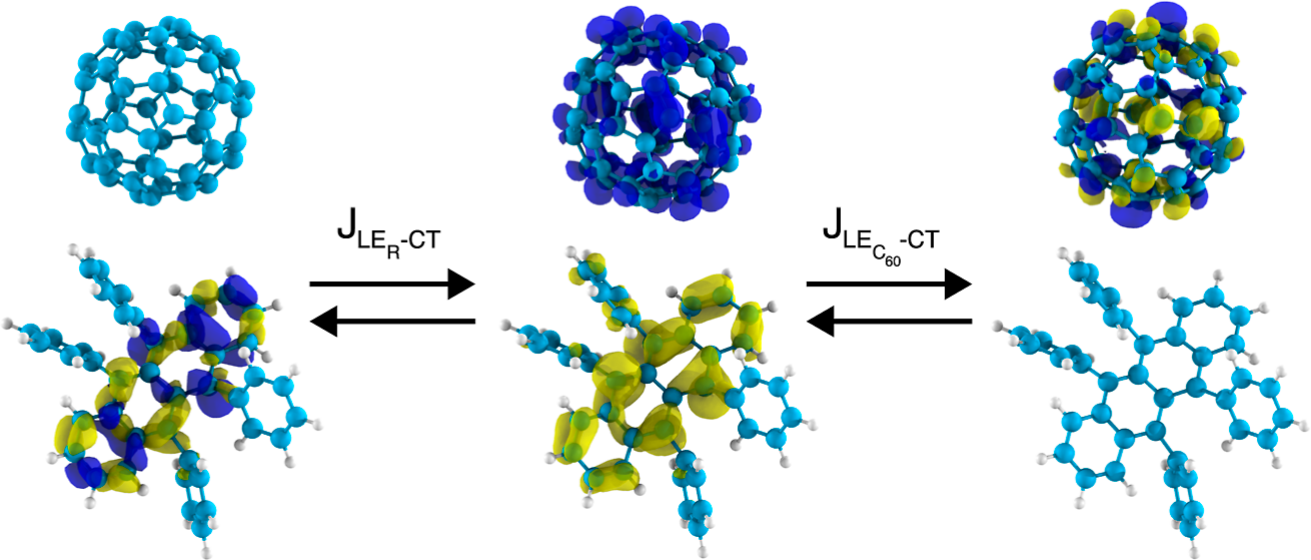

We investigate the determination of electronic coupling between localized excitations (LEs) and charge-transfer (CT) excitations based on many-body Green’s functions theory in the *GW* approximation with the Bethe–Salpeter equation (*GW*-BSE). Using a small molecule dimer system, we first study the influence of different diabatization methods, as well as different model choices within *GW*-BSE, such as the self-energy models or different levels of self-consistency, and find that these choices affect the LE-CT couplings only minimally. We then consider a large-scale low-donor morphology formed from rubrene and fullerene and evaluate the LE-CT couplings based on coupled *GW*-BSE-molecular mechanics calculations. For these disordered systems of bulky molecules, we observe differences in the couplings based on the Edmiston–Ruedenberg diabatization compared to the more approximate Generalize Mulliken–Hush and fragment charge difference diabatization formalisms. In a kinetic model for the conversion between LE and CT states, these differences affect the details of state populations in an intermediate time scale but not the final populations.

## 1 Introduction

Many photochemical processes, such as catalytic processes or the generation of charges in active layer heterostructures of organic solar cells, involve the transfer of an electron triggered by the absorption of a photon. Such photoinduced electron transfer reactions are typically influenced by a variety of properties, ranging from the intrinsic molecular electronic structure of the molecular building blocks of the material, the details of the local mutual arrangement of molecules, to larger scale morphological ordering. In many situations, the inherent disorder of the material systems in which the electron transfer takes place suggests the use of localized diabatic states to describe the reactions and to map the effects of the local and global environment on them. This idea has given rise to multiscale simulation approaches, in which the transport of excitations across a material is modeled as a series of bimolecular transfer events, each of them described by an effective transfer rate.^1−3^ According to Marcus theory,^4,5^ in the nonadiabatic high temperature or activated crossing limit, the rate of electronic excitation transfer between two states *X* (initial) and *Y* (final) is
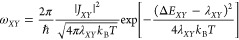
1where Δ*E*_*XY*_ is the free energy difference between the initial and final states and λ_*XY*_ is the reorganization energy. The expression also contains the electronic coupling element, *J*_*XY*_. In principle, it should be possible to evaluate all three quantities that enter the Marcus rate from electronic structure methods. To account for the local and global environment, however, it is typically required to embed electronic structure methods into a classical environment model,^6−8^ as the size of the realistic disordered systems at least in the order of several tens of nm exceeds the capabilities of explicit quantum chemistry methods. Besides such quantum-classical embedding, of the key challenges involved in the multiscale modeling approaches of this kind, it is to use quantum-chemistry methods that allow for an accurate prediction of various excited states involved in the dynamical processes. Especially for the conversion of charge-neutral excitations, e.g., after photo absorption from localized exciton (LE) to charge-transfer (CT) state as an example of a photoinduced electron transfer reaction, the energetics of both LEs and CTs need to be described on an equal footing. In this context, the use of many-body Green’s functions Theory employing the *GW* approximation and the Bethe–Salpeter equation (BSE)^9^ has become attractive to model electronically excited states on top of a ground-state reference calculation typically performed on the level of density-functional theory (DFT).^10,11^ It was shown that *GW*-BSE provides an effective single- and two-particle picture with accurate energies of LE and CT states without the need for any adaptations.^12−14^ Previous work has also shown that the additional screening caused by the molecular environment strongly affects the energies (and also densities) of CT states,^15,16^ more so than those of LEs, and that this energetic stabilization is important for finding CT-LE energy differences Δ*E*_LE-CT_ that are favorable for LE to CT conversion in organic solar cell materials. To fully treat the dynamical process of this conversion in the spirit of eq 1 requires also the reliable determination of the respective coupling elements *J*_LE-CT_.

In this work, we present a comparative study of determining electronic coupling elements between localized and CT excitations in the framework *GW*-BSE based on three different diabatization methods: Edmiston–Ruedenberg (ER) diabatization^17^ employing explicit electronic densities and the more approximate Generalize Mulliken–Hush (GMH)^18^ and fragment charge difference (FCD)^19^ formalisms. We first validate the predicted *J*_LE-CT_ in a small molecule dimer system consisting of naphthalene and tetracyanoethylene (TCNE), for which reference calculations from coupled-cluster and time-dependent DFT are available,^20^ and allow scrutinizing the individual and combined effects of energy and (effective) wave function predictions in the Green’s functions method. Herein, we also put particular emphasis on how much or little the different model choices within *GW*-BSE, such as the choice of self-energy models, different levels of self-consistency, or the use of the Tamm–Dancoff approximation (TDA) in the BSE, affect the LE-CT couplings.

To investigate how the findings for the ideal small-molecule dimer translate to larger-scale systems with potential relevance for materials applications, we proceed by applying different *GW*-BSE-based diabatization techniques to a mixed donor–acceptor blend of rubrene and fullerene.^21^ Such a blend with low-donor content contains significantly larger molecules, exhibits substantial positional and orientational disorder, and allows therefore also a case study of the kinetics of the conversion from a photoexcited LE on rubrene to rubrene-fullerene CT states, typical intermediates for charge separation.

This paper is organized as follows: in Section 2, we provide a brief summary of the essentials of the *GW*-BSE method methodology, polarizable embedding approaches, as well as the three different diabatization methods used in this work. Results on the model naphthalene-TCNE dimer and the mixed donor–acceptor system of rubrene and fullerene are presented and discussed in Section 3. A short summary concludes the paper.

## 2 Methodology

Here, we briefly summarize the essentials of many-body Green’s functions Theory in the *GW* approximation with the BSE for the calculations of electronic excitations, its polarizable embedding, as well as the three diabatization methods we consider in this work.

### 2.1 Electronic Excitations via *GW*-BSE

In the framework of *GW*-BSE,^9,22^ excitations are constructed based on a reference ground state calculation, here at the level of Kohn–Sham (KS) DFT. One first obtains KS wave functions  and energies ε_*n*_^KS^ from
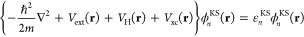
2here, *V*_ext_ is the external potential, *V*_H_ is the Hartree potential, and *V*_xc_ is the exchange–correlation potential. Hedin^23,24^ introduced the *GW* approximation of many-body Green’s functions theory, in which quasi-particle (QP) states representing independent electron and hole excitations are found as solutions in the QP equations
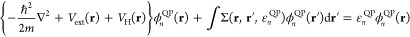
3In place of the exchange–correlation potential in eq 2, the energy-dependent self-energy operator Σ(**r**, **r**′, *E*) occurs in the QP equations. This operator is evaluated using the one-body Green’s function in QP approximation
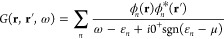
4as
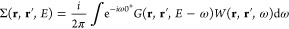
5where *W* denotes the dynamically screened Coulomb interaction. This is determined by first computing the polarization *P* in the random-phase approximation (RPA)^25,26^ and then with it the microscopic dielectric function as a convolution of *P* with the bare Coulomb interaction *v*, i.e., ϵ = 1 – *vP*. Finally, *W* is obtained as *W* = ϵ^–1^*v*, i.e., after inversion of ϵ and subsequent convolution with the bare Coulomb interaction. The frequency integration in eq 5 can be performed fully analytically based on contour deformation techniques or with the use of a generalized plasmon-pole model (PPM),^27^ which extends the RPA result for ω = 0 (static polarization) and the associated static dielectric function to the dynamic one.

Assuming that |ϕ_*n*_^QP^⟩ ≈ |ϕ_*n*_^KS^⟩, the quasiparticle energies can be evaluated perturbatively according to
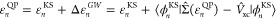
6

As the correction Δε_*n*_^*GW*^ itself depends on ε_*n*_^QP^, eq 6 needs to be solved self-consistently. In the *G*_0_*W*_0_ approximation, the single-particle energies that enter the RPA calculation of ϵ^–1^ and *G* are ε_*n*_^KS^. Updating these energies self-consistently with the corrections from eq 6 leads to the so-called eigenvalue self-consistent ev*GW* variant.

Neutral excitations with a conserved number of electrons can be obtained from the BSE.^24,28^ It determines the four-point density response function of the interacting system from the noninteracting system.^7,9,29^ Coupled electron–hole amplitudes of excitation *S* can be expressed in a product basis of QP wave functions, i.e.
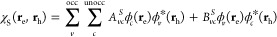
7where **r**_e_ (**r**_h_) is for the electron (hole) coordinate, and we drop the label QP for clarity. Here, *A*_*vc*_ and *B*_*vc*_ are the expansion coefficients of the excited state wave function in terms of resonant (antiresonant) transitions between QP occupied (occ.) states *v* and unoccupied (unocc.) *c*, respectively. In this basis, the BSE turns into an effective two-particle Hamiltonian problem of the form
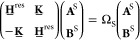
8Specifically for singlet excitations, the matrix elements of the blocks  and **K̲** are calculated as
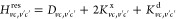
9
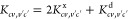
10with
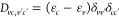
11
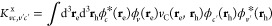
12
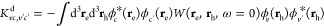
13here, *K*^x^ is the repulsive exchange interaction originating from the bare Coulomb term *v*_C_, while the direct interaction *K*^d^ contains the attractive, but screened, interaction *W* between the electron and hole, causing the binding of the electron–hole pair. In 13 it is assumed that the dynamic properties of *W*(ω) are negligible, and the computationally less demanding static approximation ω = 0 is employed. If off-diagonal blocks **K̲** in eq 8 are small, the additional use of the TDA^30^ is convenient, in which the electron–hole amplitude is expressed only as resonant transitions from occupied *v* to unoccupied *c* states
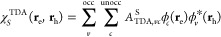
14thereby reducing the effective Hamiltonian Tothe upper diagonal block in eq 8
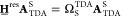
15

For all practical *GW*-BSE calculations in this work, we use the Gaussian-type orbitals implementations in the VOTCA-XTP^7,8^ software.

### 2.2 Polarizable Embedding

To account for the effects of electronic excitations in a complex molecular environment, a quantum (QM) region with the excited state complex is embedded in a classical, polarizable atomistic (MM) model for the environment. The QM/MM scheme in VOTCA-XTP makes use of a distributed atomic multipole representation for molecules in the MM region, which allows treatment of both the effects of static electric fields and the polarization response as a self-consistent reaction field. Specifically, this classical MM energy for the system is evaluated as
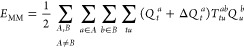
16where *A* and *B* indicate individual molecules in the system, *a* and *b* atoms in the respective molecules, *Q*_*t*_^*a*^ is the static atomic multipole moment of rank *t* associated with atom *a*, and *T*_*tu*_^*ab*^ is the tensor describing the interactions between the multipoles moments *Q*_*t*_^*a*^ and *Q*_*u*_^*b*^.^31^ The induced moment Δ*Q*_*t*_^*a*^ is generated by the electric field created by moments *t*′ of atom *a*′ ≠ *a* in molecule *A* and the one generated by the moment *u* of atom *b* in molecule *B*
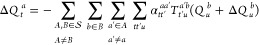
17with  the isotropic atomic polarizability on each site. To avoid the effects of spurious overpolarization, a damped version of the interaction tensor (Thole damping^31^) is used. Then, the static and induced multipoles in the MM region also interact with the electron density in the QM region via an additional external potential in eq 2. At the same time, the explicit electrostatic field from the QM density is included in polarizing the MM region. The total density of excited state *S* is evaluated from the excited-state wave function χ^S^ as
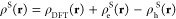
18with
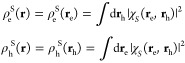
19

In order to obtain the polarization response of both the QM and MM regions, a self-consistent procedure is employed. At step *p* of this procedure, the total energy of the coupled QM/MM system for the state *S* of interest (ground state *S* = 0, or excited states *S* > 0) is determined as
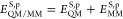
20with
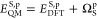
21and Ω_S_^p^ = 0 for the ground state case. The whole procedure is repeated until the change of the total energy is less than the preselected accuracy, typically 10^–5^ Ha. The excitation energy Ω_S_^QM/MM^ of a complex in the polarizable environment is then obtained as the difference
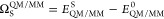
22

### 2.3 Diabatization Methods

Electronic states obtained from as eigenstates of some (approximate) Hamiltonian are adiabatic states |Φ_*i*_⟩, such as the excitations χ_*S*_ obtained from the BSE, as introduced in Section 2.1. Corresponding diabatic states |Φ_*a*_^diabatic^⟩, needed for the evaluation and understanding of electron transfer processes, can be found via a unitary transformation
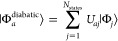
23

The unitary transformation matrix **U̲** is determined by extremalizing some function , and various methods differ by the definition of this function, with some choices being discussed below. With this, the adiabatic form of the electronic Hamiltonian *H*_el_ with adiabatic energies ε_*i*_, i.e., ⟨Φ_*i*_|*H*_el_|Φ_*j*_⟩ = ε_*j*_δ_*ij*_, is transformed into the diabatic form
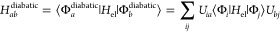
24For the two-state problem (*N*_states_ = 2), the transformation can be written explicitly as a rotation
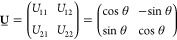
25and the diabatic Hamiltonian as
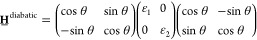
26Its off-diagonal elements
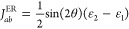
27are then the couplings between the two diabatic states.

#### 2.3.1 Edmiston-Ruedenberg Diabatization

In the ER-localized diabatization formalism,^32^ the objective is the maximization of the self-repulsion of the diabatics via
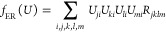
28here, the tensor *R*_*jklm*_ is defined on the basis of molecular orbitals as
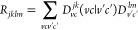
29with
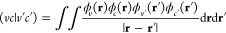
30where the indices *v* and *v*′ and *c* and *c*′ spanning the occupied and unoccupied levels, respectively. In eq 29,  is the excited state transition density matrix between the excited states *j* and *k*. If the ϕ_*n*_(**r**) is expressed in an atomic orbital basis {χ_α_(**r**)} according to , with *d*_α_^*n*^ the basis-set expansion coefficients of the molecular orbital *n* in the atomic orbital α, eq 29 can be rewritten as
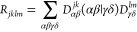
31

The tensor (αβ|γδ) is part of the standard implementation of DFT-*GW*-BSE, in which the transition density matrix between states *j* and *k* in the atomic orbital basis reads
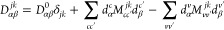
32where
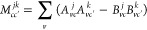
33and
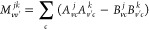
34

With these definitions, eq 31 can be computed and the ER functional can be maximized. For the two-state case, there is a closed form for this maximizing angle.^17^ It is computed with the help of
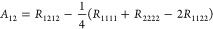
35
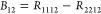
36as
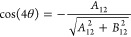
37

#### 2.3.2 Generalized Mulliken–Hush Diabatization

In the GMH approach to diabatization,^18,33^ the adabatic-to-diabatic transformation is based on the definition of the diabatic states as eigenstates of the dipole moment. Specifically in a two-state model, the method requires the calculation of the dipole moment of each adiabatic state μ_1_ and μ_1_ and the transition dipole moment between the two, μ_12_. The nonadabatic coupling element is then calculated as
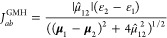
38where  is the projection of μ_12_ in the CT direction (μ_1_ – μ_2_)/|**μ**_1_ – μ_2_|.

#### 2.3.3 FCD Diabatization

Another alternative is to determine the diabatic states as eigenstates of the so-called FCD matrix,^19^ based on the definition of donor (D) and acceptor (A) fragments, as Δ*Q*_*ij*_ = *Q*_*ij*_(*D*) – *Q*_*ij*_(*A*). The fragment charges are typically obtained from a population analysis of the individual adiabatic densities and the transition density between them. Again, for a two-state model, the coupling is given by
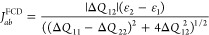
39

## 3 Results

### 3.1 Naphthalene-TCNE Complex

**Figure 1:**
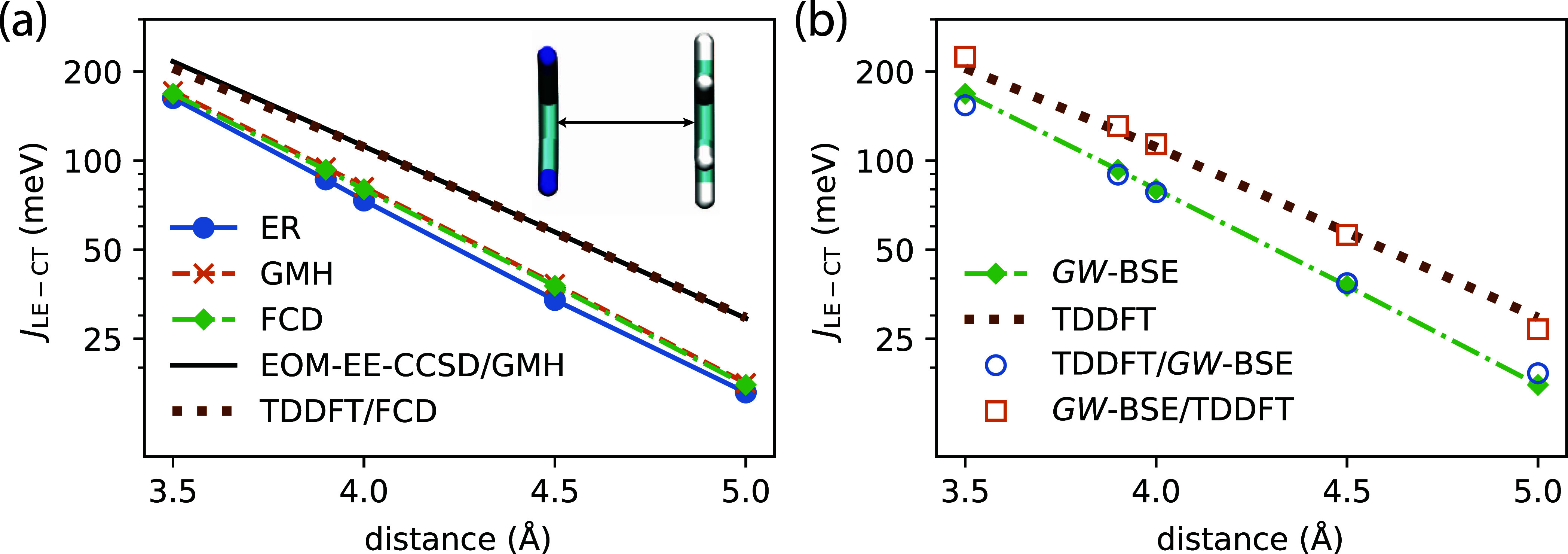
Distance dependence of LE-CT coupling elements in the naphthalene-TCNE complex. (a) *GW*-BSE results with the ER, GHM, and FCD diabatization methods based on full BSE solutions following ev*GW* calculations with FAA frequency integration, employing the def-tzvp basis and PBE0 functional in the ground state DFT run. Reference results on EOM-EE-CCSD and TDDFT levels are taken from Mao et al.^20^ The inset shows a sketch of the dimer structure. (b) Pure *GW*-BSE and TDDFT results based on FCD as in (a) compared to mixed methods, in which the fragment charge factor is taken from TDDFT and the energy difference from *GW*-BSE (TDDFT/*GW*-BSE) and vice versa, showing that the difference between the pure *GW*-BSE and TDDFT results originate from the different predicted energies.

Stacked geometries of naphthalene and TCNE, indicated as the inset of Figure 1a, with different intermolecular distances taken from ref (20). The equilibrium distance is 3.9 Å. Ground state calculations on the KS-DFT level are performed with the ORCA^34^ package using both the PBE0 functional^35^ and the def2-tzvp basis^36^ together with optimized auxiliary basis sets^37^ in resolution-of-identity techniques to efficiently express terms involving four-center Coulomb integrals. We compare in the following the results based on *G*_0_*W*_0_ calculations and eigenvalue self-consistent ev*GW* calculations. The convergence limit for the self-consistent *GW*-cycles in the ev*GW* scheme was set to 10^–5^ hartree (0.27 meV). Quasiparticle corrections are determined for the 197 lowest energy orbitals, and the product basis for the electron–hole wave functions are formed from the 66 occupied and 131 lowest unoccupied orbitals. All 1380 orbitals are included in the RPA step for calculating the dielectric function. The choice of a large range of included orbitals for small molecule systems ensures that the obtained excitation energies are safely converged to below 0.01 eV.^7,8^ Both, the fully analytic approach (FAA) and a generalized PPM, as introduced in Section 2.1 are used for the frequency integration of the self-energy. The obtained excitation energies for all variants are summarized in Table 1.

**Table 1:** Distance Dependence of the Low-Energy LE and CT Excitation Energies (in eV) in a Naphthalene-TCNE Complex, Based on Different Variants of *GW*-BSE Using the def2-tzvp Basis Set and the PBE0 Functional in the DFT Ground State Calculation

	FAA				PPM			
	ev*GW*		*G*_0_*W*_0_		ev*GW*		*G*_0_*W*_0_	
	Full	TDA	full	TDA	full	TDA	full	TDA
LE Energy								
3.5 Å	4.309	4.341	3.998	4.022	4.255	4.262	4.006	4.035
3.9 Å	4.306	4.340	4.000	4.024	4.266	4.306	4.007	4.037
4.0 Å	4.306	4.339	4.000	4.024	4.264	4.305	4.006	4.036
4.5 Å	4.305	4.339	3.997	4.022	4.262	4.303	4.003	4.033
5.0 Å	4.300	4.333	3.995	4.020	4.257	4.299	4.000	4.030
CT Energy								
3.5 Å	2.214	2.220	1.875	1.880	2.255	2.261	1.976	1.982
3.9 Å	2.387	2.389	2.052	2.054	2.430	2.432	2.157	2.159
4.0 Å	2.424	2.426	2.090	2.092	2.467	2.469	2.195	2.197
4.5 Å	2.589	2.590	2.255	2.255	2.632	2.632	2.360	2.361
5.0 Å	2.727	2.727	2.396	2.396	2.770	2.770	2.501	2.501
LE-CT Difference								
3.5 Å	2.095	2.121	2.123	2.141	2.000	2.001	2.030	2.053
3.9 Å	1.920	1.951	1.948	1.970	1.836	1.874	1.850	1.878
4.0 Å	1.881	1.913	1.909	1.932	1.797	1.836	1.811	1.839
4.5 Å	1.716	1.749	1.743	1.767	1.630	1.670	1.642	1.673
5.0 Å	1.572	1.606	1.599	1.623	1.488	1.529	1.499	1.529

In Figure 1a, we show the distance-dependent LE-CT couplings resulting from ev*GW*-BSE calculations with the FAA, the def2-tzvp basis set, and PBE0 in the ground state calculation. We first compare the influence of the choice of the diabatization method, with the couplings obtained from ER shown as circles, from GMH as crosses, and FCD as diamonds. While both GMH and FCD methods appear to yield very similar couplings with a maximum deviation of 4 meV at a separation of 3.5 Å (see also Table 2), the *J*_LE-CT_^ER^ values result slightly lower, e.g., by 9 meV at the closest distance. Note that the respective slopes of the three *GW*-BSE based data are identical, reflecting the same exponential decay of the LE-CT coupling with distance. Comparing out the results with those obtained by EOM-EE-CCSD/GMH and TDDFT/FCD with the ωB97X-D^38^ functional^20^ shown in Figure 1a as solid and dashed lines, respectively, we observe a combination of an offset to lower energies and a slightly stronger slope in *GW*-BSE. For instance, at the optimal intermolecular distance of 3.9 Å, EOM-EE-CSSD/GMH predicts a LE-CT coupling of 128 meV, TDDFT/FCD 126 meV, compared to 86 meV from ER, 95 meV from GMH, and 93 meV from FCD with *GW*-BSE. To understand this difference, we take a closer look at the results obtained with FCD diabatization in Figure 1b. The *GW*-BSE and TDDFT results from Figure 1a are shown again, now combined with “mixed” versions. In these versions, we first distinguish in the expression for *J*_LE-CT_^FCD^ between the fragment charge contribution *f*_FCD_ = *J*_LE-CT_^FCD^/(ε_2_ – ε_1_) and the energy contribution *f*_ε_ = (ε_2_ – ε_1_). Then, we combine *f*_FCD_(TDDFT) with *f*_ε_(*GW*-BSE) (TDDFT/*GW*-BSE) and vice versa (*GW*-BSE/TDDFT). For the former, we find that the resulting couplings are essentially identical to the ones from pure *GW*-BSE, while the latter results are in close agreement with the full pure TDDFT data. This corroborates the notion that the difference between the pure TDDFT and *GW*-BSE derived couplings can to a large extend be attributed to differences in the energies. From Table 1, the LE-CT energy difference at the optimal naphthalene TCNE distance is 1.92 eV in *GW*-BSE and 2.70 eV in TDDFT,^20^ and their ratio almost exactly translates into the ratio of the respective coupling elements. The observed difference of the LE-CT energy splitting from *GW*-BSE and TDDFT can be attributed mostly to known issues with obtaining accurate CT excitation energies in TDDFT without tuned range-separated functionals.^39^ Notably, in EOM-EE-CCSD, this energy split is reported as 1.47 eV, which would indicate an even smaller LE-CT coupling than in *GW*-BSE based on *f*_ε_ only. Here, there are more differences in the factors related to the transitions in terms of dipole moments or fragment charges.

**Table 2:** LE-CT Coupling Elements (in meV) in the Naphthalene-TCNE Complex at Several Intermolecular Distances, as Obtained Using ER, GMH, and FCD Diabatization with Different Variants of *GW*-BSE Using the def2-tzvp Basis Set and the PBE0 Functional in the DFT Ground State Calculation

	FAA				PPM			
	ev*GW*		*G*_0_*W*_0_		ev*GW*		*G*_0_*W*_0_	
	full	TDA	full	TDA	full	TDA	full	TDA
ER Diabatization								
3.5 Å	163	165	158	161	161	163	155	155
3.9 Å	86	87	84	85	88	89	86	86
4.0 Å	73	74	71	72	75	76	73	73
4.5 Å	34	34	33	33	35	35	34	34
5.0 Å	16	17	16	17	17	18	17	17
GMH Diabatization								
3.5 Å	172	172	169	169	169	169	165	165
3.9 Å	95	95	93	94	93	93	92	92
4.0 Å	81	81	80	80	80	80	79	79
4.5 Å	38	38	38	38	38	38	37	37
5.0 Å	18	18	18	18	17	17	17	17
FCD Diabatization								
3.5 Å	168	168	165	166	165	165	161	162
3.9 Å	93	93	92	92	92	92	90	90
4.0 Å	80	80	79	80	79	79	77	77
4.5 Å	38	38	37	37	37	37	37	37
5.0 Å	17	17	17	17	17	17	17	17

Table 2 also contains LE-CT couplings as obtained from the different variants of *GW*-BSE, in which we have changed the exact frequency integration in eq 5 with a PPM, the level of *GW* from ev*GW* to *G*_0_*W*_0_, and/or the BSE from its full form to the TDA. Overall, the *J*_LE-CT_ values are not very sensitive to the specific choices in the *GW* and BSE steps. For the sake of clarity, we will focus on the results from ER diabatization at the optimal separation of 3.9 Å in the following. First, the use of the TDA of the BSE impacts the couplings by only 1 meV, also the use of the one-shot *G*_0_*W*_0_ method instead of ev*GW* does not show differences exceeding 3 meV. Even the use of the PPM in place of the exact frequency integration (FAA) is of the same order, so that all values are within 3% of the FAA/ev*GW*/full BSE result. Similar observations also hold for the other intermolecular distances and diabatization techniques.

### 3.2 Rubrene-Fullerene Low-Donor Content System

We now move from the well-ordered, small molecule naphthalene-TCNE dimer to a disordered cluster of larger molecules and investigate the sensitivity of LE-CT coupling elements based on *GW*-BSE on the different diabatization methods and if eventual differences propagate to different answers in dynamic models of conversion between LE and CT states. Specifically, we study an amorphous morphology with low-donor content (<10 mol %), composed of fullerene (C_60_) and 5,6,11,12-tetraphenyltetracene (rubrene).^21^ Because of the low-donor content, a C_60_ cluster will surround the donor molecule, making the interaction between the single donor molecule with a close shell of neighboring C_60_ acceptors representative of the properties of the system as a whole. These complexes are therefore meaningful candidates for a computational analysis of the influence of donor–acceptor conformations and environment polarization effects in the *GW*-BSE/MM framework introduced in Section 2.2 and its consequence on the conversion dynamics between initially excited LE on rubrene (LE_R_) to CT excitations.

#### 3.2.1 CT Density of States

To obtain representative structures, mixed morphologies have been simulated with ab initio MD based on density functional tight binding theory using linear scaling self-consistent field calculations within the CP2K code.^40^ Initial configurations have been prepared using Packmol,^41^ targeting experimental values^21^ for densities and mole percentages. This structure is first equilibrated at 700 K in *NpT* (with velocity rescaling thermostat^42^ at atmospheric pressure^43^) for 7 ps (time step 1 fs) and then annealed to 300 K within 10 ps. A final *NpT* equilibration is followed for 5 ps.

For calculating the LE and CT densities of states, C_60_ molecules are selected which are approximately in the first neighbor shell around one rubrene molecule. Given the conformation of this low-donor content materials, the behavior of this shell of molecules should be representative of the overall behavior of the material. After selection, polarizable *GW*-BSE/MM embedding calculations, as described in Section 2.2 are performed for all dimers formed by rubrene and fullerene. Specifically, for the *GW*-BSE calculations, we employ the def2-tzvp basis set^36^ with an optimized auxiliary basis^37^ for the steps including resolution-of-identity. The ground-state DFT calculation uses the PBE0 functional.^35^

To keep the computational costs tractable, we determine the optimal number of states included in the *GW* and BSE steps by starting, for one of the dimer systems, from a small range of states around the highest occupied molecular orbital (HOMO)–lowest unoccupied molecular orbital (LUMO) gap and to systematically increasing the ranges until we obtain LE and CT excitation energies converged to within 0.01 eV. As a result, eigenvalue self-consistent *GW* (ev*GW*) calculations are performed to obtain the explicit quasiparticle-corrected energies for the highest 100 occupied and lowest 100 unoccupied orbitals, respectively. All orbitals are included in the RPA step and not explicitly *GW*-corrected levels are scissors shifted according to the highest absolute quasiparticle correction among the explicitly corrected occupied or unoccupied orbitals. The frequency integration in eq 5 is performed using the PPM. Coupled electron–hole wave functions according to eq 7 are constructed using transitions between the highest 220 occupied and 220 lowest unoccupied states. In the MM part of the *GW*-BSE/MM, polarizable electrostatic interactions are taken into account within a cutoff of 4 nm. As the static moments, we consider CHELPG^44^ atomic partial charges obtained from reference DFT calculations on rubrene and C_60_ monomers. Similarly, the isotropic atomic polarizabilities are optimized such that the volumes of the molecular polarizability tensors in the classical representation and DFT reference match.

**Figure 2:**
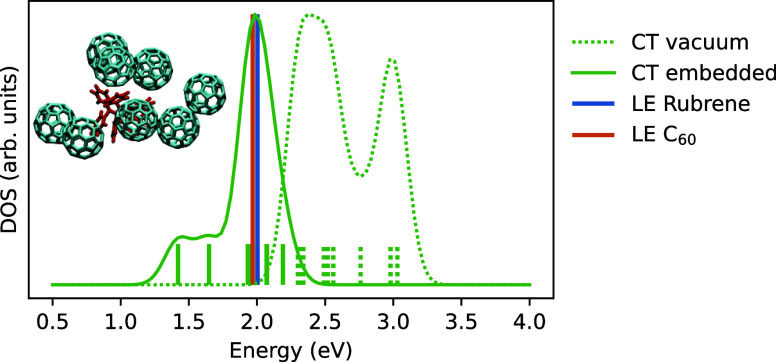
Energies of CT excitations (green vertical bars) from vacuum *GW*-BSE (dashed) and *GW*-BSE/MM calculations with polarizable embedding (solid). Solid and dashed curves indicate respective density-of-states obtained by Gaussian broadening with 0.1 eV as a guide-to-the-eye. Blue and orange lines highlight the *GW*-BSE/MM energies of LEs on rubrene and C_60_, respectively.

The resulting energies of CT and LE excitations are depicted in Figure 2. In general, the effects of polarizable embedding on the LE energies are small, as has been observed before, e.g., for embedded push–pull polymers^15^ or small-molecule donor molecules.^8^ Therefore, we only show the *GW*-BSE/MM results for the respective LEs, indicated by the blue (rubrene at 2.01 eV) and orange (C_60_ at 1.97 eV) vertical lines as there is no noticeable disorder. For the CT excitations, the *GW*-BSE calculations in vacuum already reveal significant energetic disorder, originating from the different rubrene-C_60_ conformations and the long-range electrostatic interaction between electron and hole. Individual CT excitation energies are marked by the short green vertical lines in Figure 2, where we also show a density-of-states obtained by broadening with a Gaussian function of width 0.1 eV. After polarizable embedding in *GW*-BSE/MM, the CT energies (solid green lines) are shifted to lower energies, with energetic stabilization of up to 1 eV. Note that in vacuum, the CT excitation energies result generally higher than both LEs, which would make the conversion process of a LE on rubrene to a CT state energetically unlikely. After embedding, we find that the energetic stabilization brings several high-energy CTs close to the LEs and some notably very much lower at 1.65 eV (CT_1_) and 1.42 eV (CT_0_). The latter compares favorably with the experimentally measured CT energy of 1.46 eV, as reported in ref (21). However, given the disorder in the CT excitation energies, it is unclear if the measurement truly probes simply the lowest-energy CT state or one that is preferably dynamically populated during the time scale of the conversion process and the experiment.

#### 3.2.2 Electronic LE-CT Couplings

To proceed beyond considering only the energy difference for the conversion of LE to CT excitons, we consider the LE-CT couplings and analyze if the disorder in them could be indicative of some dimers not participating in the process. We also investigate if for such large bimolecular structures, the use of GHM, FCD, and ER diabatization has any influence on the results.

**Figure 3:**
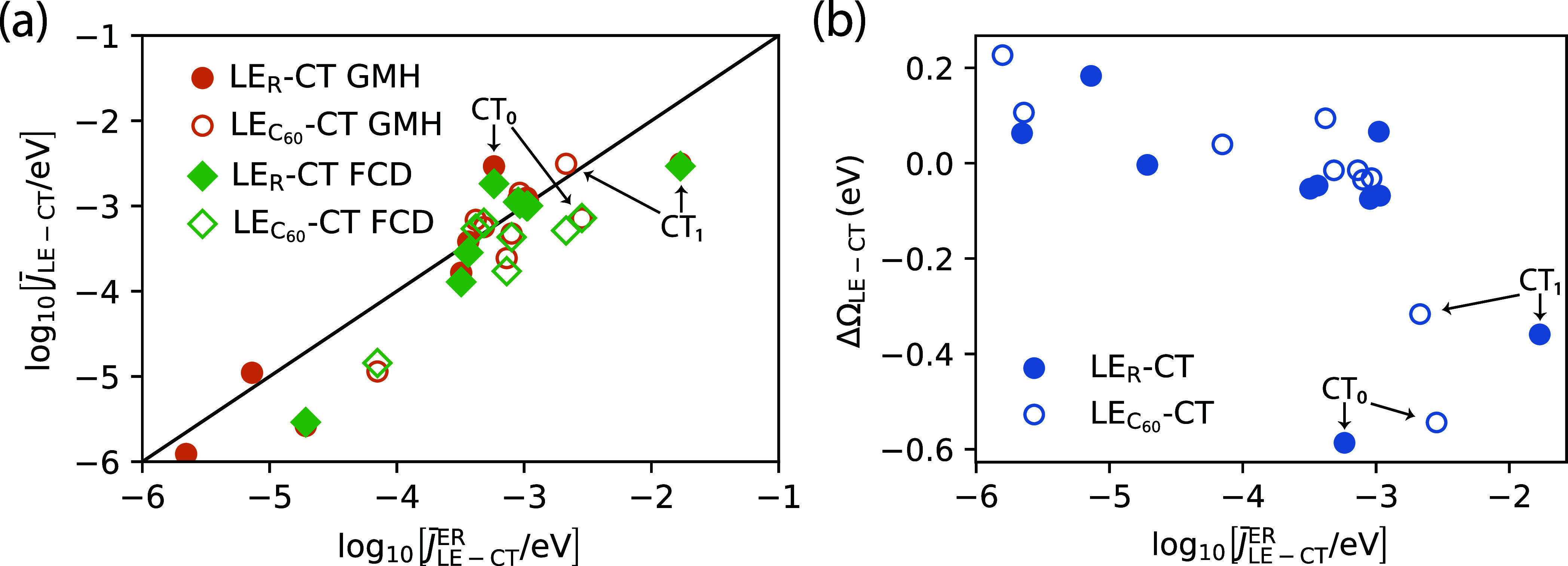
(a) Effective LE-CT couplings (see eq 40) in the rubrene-C_60_ dimers resulting from polarizable *GW* – BSEmm calculations with the GMH and FCD diabatization schemes against those from ER. (b) Relation between LE-CT energy difference ΔΩ_LE-CT_ = Ω_CT_ – Ω_LE_ and the LE-CT coupling from *GW*-BSE ER diabatization.

A specific aspect of the rubrene-C_60_ systems that requires extra is the (near) degeneracy of the 15 lowest LE on C_60_, stemming from the 5-fold degeneracy of its HOMO and 3-fold degeneracy of its LUMO. For the same reason, also the CT states are 3-fold near degenerate. We take this into account by calculating an effective diabatic coupling^45,46^ between *N*_LE_-fold degenerate LEs and *N*_CT_-fold degenerate CT excitons as
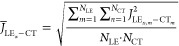
40with  the coupling element between the *m*-th degenerate LE and the *n*-th degenerate CT. The results from the different diabatization methods are shown in Figure 3a. There, we plot the couplings obtained with GMH and FCD diabatization against those from ER and distinguish between LE_R_-CT (filled symbols) and LE_C_60__-CT (open symbols) couplings. Roughly speaking, the effective couplings from ER cover a range from 0.02 to 17 meV, with many occurring close to 1 meV. Compared to the small-molecule naphthalene-TCNE dimer with ideal stacking, we find a stronger dependence on the diabatization method, although the differences between GMH and FCD seem minor in most cases. Of particular interest are the couplings of the two low-energy CT states, CT_0_ and CT_1_, as marked in Figure 3a. Specifically, the LE_R_-CT couplings are different using ER (0.6 vs 17 meV), while they are similar when using GMH at about 3 meV. As the ER method takes the full details of the electronic (transition) densities into account, it stands to reason that the extra details have a bigger contribution to the LE-CT couplings for more disordered structures and larger molecular building blocks than in ordered clusters with additional symmetry. This notion is corroborated by the observation that for the rubrene-C_60_ system similar differences between GHM and ER couplings are found also for the respective dimers in vacuum, which excludes the possibility that they are attributable to environment effects, instead.

In Figure 3b, we show the relation between the energy offset of LE and CT states, calculated as ΔΩ_LE-CT_ = Ω_CT_ – Ω_LE_, and the LE-CT couplings obtained with ER. From 27 one generally expects some dependence of the couplings on the energy difference. Some dependence is visible in Figure 3b, although it is hard to ascribe a definite trend to the data. Noteworthy is that the two dimers with the most negative energy offsets correspond to the two low energy CT states, as discussed in Section 3.2.1. In particular, CT_1_ at energy 1.65 eV is found to have the highest coupling between the rubrene LE and the CT state. In comparison, the coupling to the lowest CT state, CT_0_, is smaller by a factor of 30. This raises the question what impact the differences in couplings have for the LE-CT conversion dynamics, particularly of which the CT states is most likely to be probed over which time scale.

#### 3.2.3 Kinetic Model

To scrutinize the effects of the disorder in energies and LE-CT couplings obtained from the *GW*-BSE/MM calculations in Sections 3.2.1 and 3.2.2 and the influence of different diabatization methods, we now study the conversion dynamics between LE and CT excitations with a kinetic model based on Marcus rates as in eq 1. This model requires in addition to the calculation of the LE-CT couplings and the excitation energies Ω also the determination of the respective reorganization energies λ_*ab*_. Within the Marcus picture,  and λ_CT-LE_*x*__ = *E*_LE_*x*__(CT)-*E*_LE_*x*__(LE_*x*_), where *x* = R,C_60_ and *E*_*a*_(*G*) represent the total energy of state *a* at geometry of state *G*. As such, this would require the cumbersome optimization of the dimer structures in the respective CT and LR states. Instead, we approximate the energies from monomer calculations, such that
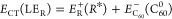
41
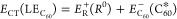
42
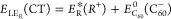
43
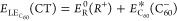
44
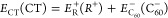
45
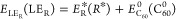
46
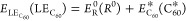
47where the superscripts refer to the state of the monomers (0: ground state, +: cation, −: anion, *: excited). The total energy calculations and geometry optimizations in this step are performed using (time-dependent) DFT with the same basis set and functional as the *GW*-BSE calculations in Section 3.2.1, and we obtain  0.12 eV,  0.12 eV,  = 0.18 eV, and  = 0.21 eV. In similar spirit, we determine the vertical to adiabatic energy relaxations of the excited states, Λ_*a*_ = *E*_*a*_(0) – *E*_*a*_(*A*), as Λ_CT_ = 0.15 eV, Λ_LE_R__ = 0.16 eV, and  0.23 eV, needed to convert the vertical excitation energies Ω obtained from *GW*-BSE to adiabatic energies *E* needed in the Marcus rate eq 1.

With all the energies and coupling elements at hand, we determine all rates between LE and CT states according to eq 1 at *T* = 300 K for the kinetic model, which describes the time-evolution of the state population probabilities **P**(*t*) via a system of ordinary differential equations of the kind
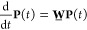
48

**Figure 4:**
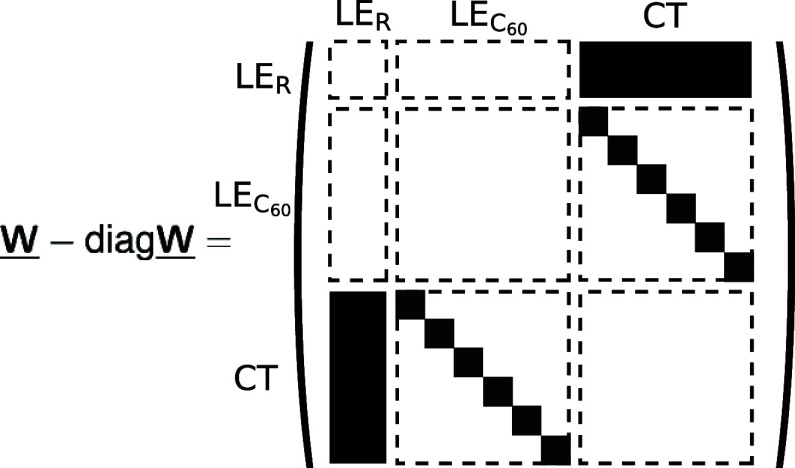
Schematic representation of the rate matrix of a kinetic model, with full squares indicating the respective rates at which LE-CT couplings occur.

In this specific case, **P**^*T*^(*t*) = [*P*_LE_R__(*t*), ] is of dimension 21, and ∑_*i*_*P*_*i*_(*t*) = 1 for all *t*. The structure of the off-diagonal entries of the matrix **W̲** is shown in Figure 4, which correspond to the respective Marcus rates according to eq 1, emphasizing again that in this minimal model, we only consider transitions between LE and CT states and not between different LE and different CTs. The diagonals of **W̲** contain the negative of the sum of all other column entries, i.e., *W*_*ii*_ = −∑_*j*_*W*_*ji*_.

**Figure 5:**
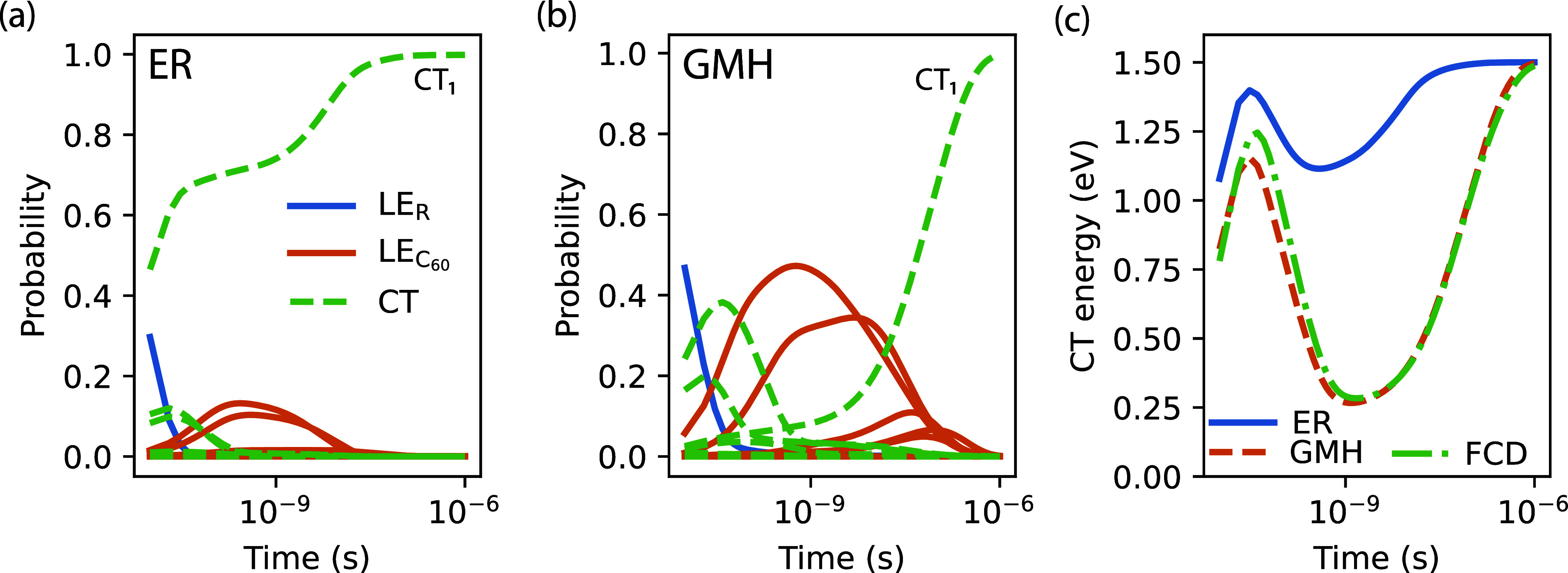
(a) Population dynamics of the excited states for *t*_max_ = 1 μs as a solution to eq 48 for LE-CT couplings from ER diabatization. The blue line indicates the population of the LE_R_ excited state, orange lines indicate the ones of the respective LE_C_60__, and green dashed lines indicate the populations of CT states. (b) Same for GMH diabatization. (c) Time evolution of the expected CT energy ⟨*E*_CT_⟩ from population probabilities based on models with different diabatization methods.

We initially prepare the system in the LE_R_ state, i.e., **P**^*T*^(*t* = 0) = [1, 0, ... , 0], and numerically study the evolution of eq 48 for *t*_max_ = 1 μs using the backward Euler scheme^47^ with 10^5^ steps. In Figure 5a, we show the resulting population probabilities with the LE-CT couplings calculated using ER diabatization. Initially, the population of the LE_R_ state decays rapidly, and it is completely depopulated within 50 ps. This initial decay occurs primarily into three CT states, with a clear preference for the CT_1_ state. After 50 ps, the two additionally populated CT states convert first into LE_C_60__ as intermediates in the time scale of 100 ps to 10 ns, until they also decay nearly exclusively into CT_1_. Note that we do not observe over the time scale of 1 μs, a noticeable population of the lowest energy CT state, CT_0_. As can be seen from Figure 5b, there are some qualitative similarities when the dynamics are modeled based on GMH diabatization. In particular, the same rapid initial decay of LE_R_ and the final near complete population of CT_1_ can be seen. Some quantitative difference can be noted in the details of the intermediate dynamics. Initially, CT_1_ does not get populated. Instead, the populations of the two other CT states is much higher and, consequently, also the populations of the two intermediate LE_C_60__ they convert into.

We also report in Figure 5c the expectation value of the adiabatic CT energy, calculated according to
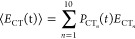
49for the three different diabatization methods considered in this work. As could be expected from the individual populations over time, the expected CT energy follows similar trends. At about 50 ps, all methods exhibit a peak, whose height depends slightly on the method. Its energy of more than 1 eV is, however, a consequence of the low population of high energy (∼2 eV) CT states and not indicative of the population of either CT_0_ or CT_1_. The dip following the peak is a combined effect of the depopulation of the high-energy CT states to both CT_1_ (in case of ER) and some LE_C_60__. In the model based on GMH/FCD, the cumulative population of all CT states is minimal at *t* = 1 ns, leading to the pronounced reduction of the expected CT state energy. On the other hand, in the ER model, the CT_1_ state is already populated at this time, but the combination of it being a low energy excitation and only partially populated (around 0.6) still leads to a smaller but noticeable minimum. Only on the time scale of 1 μs, when the CT_1_ population is nearly 1, ⟨*E*_CT_(*t*)⟩ is indicative of a pure CT state. Interestingly, the value of ⟨*E*_CT_(*t* = 1 μs)⟩ = 1.50 eV is close to the CT energy reported in experiments (1.46 eV),^21^ although we do not want to overstress this apparent agreement due to the limited nature of the model. Noteworthy in this context is also that in both cases (ER and GMH/FCD), no population of CT_0_ is observed in the considered time scale. This is a combined effect of the significantly smaller coupling to CT_0_ and the property of the Marcus rate, which shows decreasing rates for large positive energetic driving forces Δ*E* > λ (“inverted regime”). In thermal equilibrium (*t* → ∞), one would expect the state occupation probabilities to be Boltzmann distributed according to
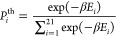
50with β^–1^ = *k*_B_*T*, and as such, an almost complete population of CT_0_. While out limited model appears to run into a different equilibrium with complete CT_1_ occupation as in Figure 5, this is misleading, as even in the model, a conversion to CT_0_ will happen on a much longer time scale (roughly milliseconds). Note, however, that adding additional conversion pathways to the model is expected to reduce the time in which the system reaches thermal equilibrium. Such additional pathways might involve direct transfer among the different CTs or LEs or the decay of excitations due to finite lifetimes. In principle, the diabatization techniques based on *GW*-BSE/MM are expected to be applicable and also the LE–LE and CT–CT electronic couplings. Alternatively, one could use dimer projection techniques^48^ which start out from LEs in monomer calculations as approximate diabatic states in the complex. These have been used, for instance, with finite lifetime estimates in large-scale simulations of singlet and triplet exciton dynamics in molecular crystals.^49^ Accounting for these processes in a kinetic model is essential for a full first-principles study of the dynamics of excitonic processes in disordered, complex molecular materials. However, including them in the present study is beyond the scope of this work which focuses on the analysis of different diabatization methods with respect to the calculation of LE-CT couplings. Finally, we comment that for the modeling of dynamical processes such as the ones in this work, it appears that the choice of the diabatization method in the calculation of LE-CT couplings is less critical for qualitative and semiquantitative insights than the proper inclusion of environment effects for the excitation energies. It is obvious from Figure 2 and eq 50 that no conversion from LE to CT would take place, if the respective excitation energies in vacuum were used.

## 4 Summary

In summary, we have developed the determination of LE-CT coupling elements withing the framework of *GW*-BSE. We have shown that in an ideal small-molecule dimer of naphthalene and TCNE, the quantitative estimates of these couplings are largely insensitive to methodological choices in the *GW* and BSE steps of the calculation, and only small differences are noted between the ER, Generalized Mulliken–Hush, and FCD diabatization formalisms. Compared to literature results for this model system on the TD-DFT level, we could show that the difference found in the *GW*-BSE-based calculations can be attributed to different predictions of the adiabatic dimer energies entering the diabatization procedure and not differences in the densities of the excitations.

In a larger scale, disordered molecular complexes, such as the low-donor content rubrene-fullerene mixtures, the LE-CT couplings are found to be more sensitive to the choice of the diabatization formalism. While the two more approximate Generalized Mulliken–Hush and FCD approaches yield couplings that are largely in agreement with each other, they differ from the respective results based on the ER approach, which takes full details of the excited state densities into account. To scrutinize the effect of the different predictions both qualitatively and quantitatively, we have employed the respective LE-CT couplings in a minimal kinetic model of the conversion from LE to CT states based on Marcus rates. From the obtained time evolution of state population probabilities, it is apparent that the dynamics are affected on an intermediate time scale but not the final steady state prediction.
